# Chaetocin Improves Pig Cloning Efficiency by Enhancing Epigenetic Reprogramming and Autophagic Activity

**DOI:** 10.3390/ijms21144836

**Published:** 2020-07-08

**Authors:** Pil-Soo Jeong, Bo-Woong Sim, Soo-Hyun Park, Min Ju Kim, Hyo-Gu Kang, Tsevelmaa Nanjidsuren, Sanghoon Lee, Bong-Seok Song, Deog-Bon Koo, Sun-Uk Kim

**Affiliations:** 1 Futuristic Animal Resource & Research Center, Korea Research Institute of Bioscience and Biotechnology, Chungcheongbuk-do 28116, Korea; spectrum@kribb.re.kr (P.-S.J.); embryont@kribb.re.kr (B.-W.S.); tngusdl30@kribb.re.kr (S.-H.P.); jmmy05@kribb.re.kr (M.J.K.); kogd1887@kribb.re.kr (H.-G.K.); tsevelmaa@kribb.re.kr (T.N.); sodany2@kribb.re.kr (S.L.); sbs6401@kribb.re.kr (B.-S.S.); 2 Department of Biotechnology, Daegu University, Gyeongsangbuk-do 38453, Korea; 3 Department of Functional Genomics, KRIBB School of Bioscience, Korea University of Science and Technology (UST), Daejeon 34113, Korea

**Keywords:** chaetocin, epigenetic reprogramming, H3K9me3, DNA methylation, porcine SCNT embryo, autophagy

## Abstract

Efficient epigenetic reprogramming is crucial for the in vitro development of mammalian somatic cell nuclear transfer (SCNT) embryos. The aberrant levels of histone H3 lysine 9 trimethylation (H3K9me3) is an epigenetic barrier. In this study, we evaluated the effects of chaetocin, an H3K9me3-specific methyltransferase inhibitor, on the epigenetic reprogramming and developmental competence of porcine SCNT embryos. The SCNT embryos showed abnormal levels of H3K9me3 at the pronuclear, two-cell, and four-cell stages compared to in vitro fertilized embryos. Moreover, the expression levels of H3K9me3-specific methyltransferases (*suv39h1* and *suv39h2*) and DNA methyltransferases (*DNMT1*, *DNMT3a*, and *DNMT3b*) were higher in SCNT embryos. Treatment with 0.5 nM chaetocin for 24 h after activation significantly increased the developmental competence of SCNT embryos in terms of the cleavage rate, blastocyst formation rate, hatching rate, cell number, expression of pluripotency-related genes, and cell survival rate. In particular, chaetocin enhanced epigenetic reprogramming by reducing the H3K9me3 and 5-methylcytosine levels and restoring the abnormal expression of H3K9me3-specific methyltransferases and DNA methyltransferases. Chaetocin induced autophagic activity, leading to a significant reduction in maternal mRNA levels in embryos at the pronuclear and two-cell stages. These findings revealed that chaetocin enhanced the developmental competence of porcine SCNT embryos by regulating epigenetic reprogramming and autophagic activity and so could be used to enhance the production of transgenic pigs for biomedical research.

## 1. Introduction

The pig is a useful animal model because of its physiological and anatomical similarities to humans [1,2]. Transgenic pigs can be produced by somatic cell nuclear transfer (SCNT) [3]. However, the cloning efficiency in terms of pre- and post-implantation embryo development is very low [4], which limits the application of transgenic pigs. Therefore, it is important to increase cloning efficiency in order to improve the production of transgenic pigs.

SCNT enables reprogramming of differentiated somatic cells into a totipotent state from a donor nucleus using an enucleated oocyte [5,6]. However, incomplete reprogramming in SCNT embryos results in a low cloning efficiency [7]. Histone H3 lysine 9 trimethylation (H3K9me3) is enriched in reprogramming resistant regions of SCNT embryos and blocks donor cell reprogramming, leading to failure of preimplantation development in mice [8], humans [9], pigs [10,11], and monkeys [12]. H3K9me3 is a histone modification marker and its levels are highly correlated with that of constitutive heterochromatin [13]. The H3K9me3-specific methyltransferases *suv39h1* and *suv39h2* are highly expressed in heterochromatin regions of mammalian cells [14]. Injection of an H3 lysine9-specific demethylase (KDM) into SCNT embryos or silencing of the expression of *suv39h1* and *suv39h2* in donor cells modulates the H3K9me3 level and so increases developmental efficiency [8,15]. In addition, previous studies reported that *suv39h1* and *suv39h2* interact directly with the DNA methyltransferases (DNMTs), including *DNMT1*, *DNMT3a*, and *DNMT3b*, to methylate DNA with heterochromatin protein 1 (HP1), a transcriptional repressor, the end result being modulation of gene transcription [16,17].

Chaetocin is a fungal mycotoxin primarily produced by *Chaetomium minutum* [18], and it has antibiotic properties and a thiodioxopiperazine structure [19]. Pharmacological inhibition of *suv39h1* and *suv39h2* by chaetocin results in reduced H3K9me3 levels [20]. Chaetocin has been reported to have anticancer activity by suppressing differentiation and proliferation in various cancer cell lines [21,22,23]. Previous studies reported that chaetocin shows antimyeloma activity by inducing oxidative stress and antihepatoma activity by dysregulating the splicing of hypoxia-inducible factor 1α [24,25]. However, few previous studies have addressed chaetocin during preimplantation embryonic development, so further studies are still needed to investigate the underlying mechanism(s) on porcine SCNT embryo development. Therefore, we investigated the optimal duration and concentration of chaetocin treatment in terms of enhancing in vitro developmental competence (blastocyst formation rate, total cell number, and cell survival rate) and changing the epigenetic reprogramming during porcine SCNT embryo development. We also investigated the effects of chaetocin on the H3K9me3 level and global DNA methylation in porcine SCNT embryos, and confirmed the effect of chaetocin on autophagic activity and the levels of maternal mRNAs in porcine SCNT embryos using immunofluorescence and quantitative real-time polymerase chain reaction (qRT-PCR).

## 2. Results

### 2.1. H3K9me3 Levels in In Vitro Fertilized and SCNT Embryos

**Figure 1:**
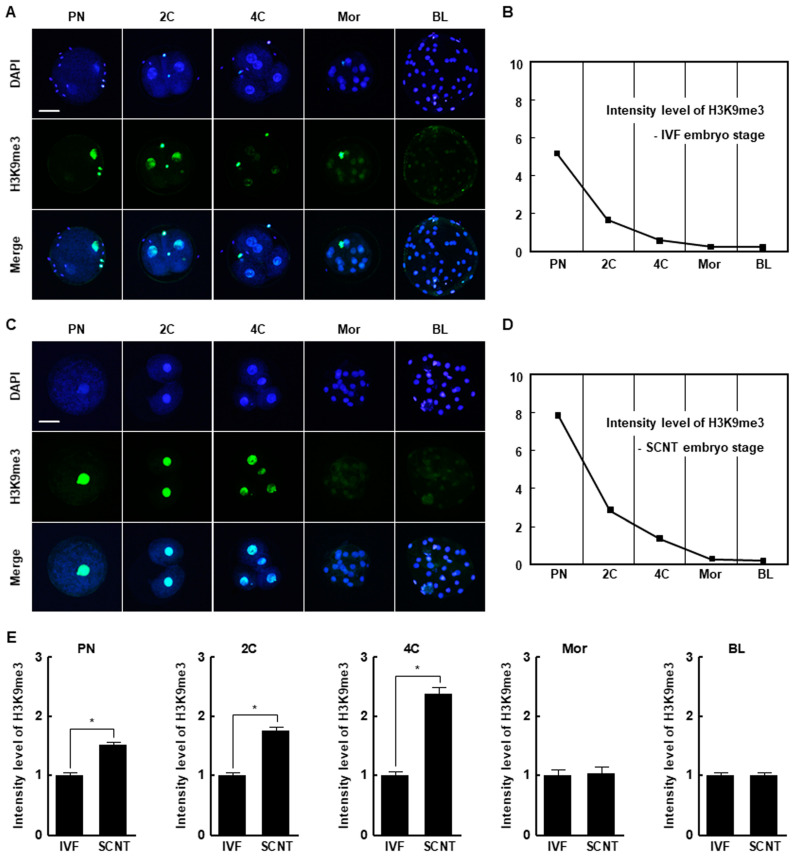
Histone H3 lysine 9 trimethylation (H3K9me3) levels in in vitro fertilization (IVF) and somatic cell nuclear transfer (SCNT) embryos during porcine preimplantation development. (**A**) Representative immunofluorescence images of H3K9me3 in IVF embryos at the indicated developmental stages. Embryos were stained for H3K9me3 (green) and DNA (4′,6′-diamidino-2-phenylindole [DAPI], blue). Bar = 50 µm. (**B**) H3K9me3 level during preimplantation development of IVF embryos (*n* = 20 per group). (**C**) Representative immunofluorescence images of H3K9me3 in SCNT embryos at the indicated developmental stages. Embryos were stained for H3K9me3 (green) and DNA (DAPI, blue). Bar = 50 µm. (**D**) H3K9me3 level during preimplantation development of SCNT embryos (*n* = 20 per group). (**E**) Quantification of H3K9me3 levels in IVF and SCNT embryos at the indicated developmental stages (*n* = 20 per group). The data are from three independent experiments and are means ± standard error of the mean (SEM) (* *p* < 0.05). PN, pronuclear stage; 2C, two-cell stage; 4C, four-cell stage; mor, morula stage; BL, blastocyst stage.

**Figure 2:**
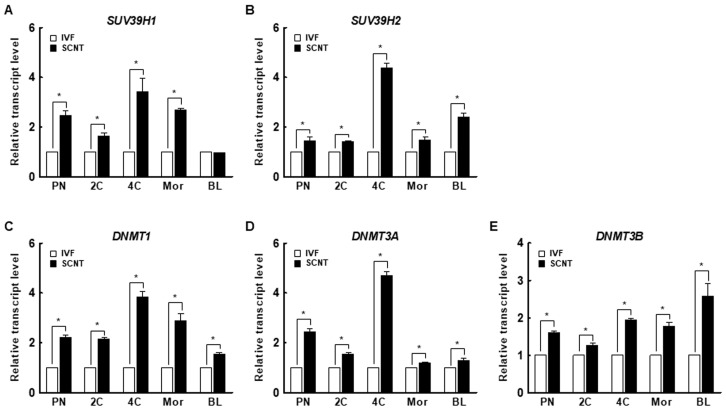
Expression of the H3K9me3-specific methyltransferases (**A**) suppressor of variegation 3-9 homolog 1 (*suv39h1*) and (**B**) suppressor of variegation 3-9 homolog 2 (*suv39h2*) and the DNA methyltransferases (DNMTs) (**C**) *DNMT1*, (**D**) *DNMT3a*, and (**E**) *DNMT3b* in IVF and SCNT embryos during preimplantation development (*n* = 3 per group). The data are from three independent experiments and are means ± SEM (* *p* < 0.05). PN, pronuclear stage; 2C, two-cell stage; 4C, four-cell stage; mor, morula stage; BL, blastocyst stage.

The H3K9me3 levels in SCNT embryos was highest at the pronuclear stage and subsequently decreased at the blastocyst stage, similar to the pattern in in vitro fertilization (IVF) embryos (Figure 1A–D). However, SCNT embryos showed a significantly higher level of H3K9me3 at the pronuclear, two-cell, and four-cell stages compared to IVF embryos, whereas there were no differences at the morula and blastocyst stages (Figure 1E). The expression levels of *suv39h1* and *suv39h2* decreased from the two-cell stage to the blastocyst stage in both IVF and SCNT embryos (Figure S1A,B), but their expression levels in SCNT embryos were significantly higher than in IVF embryos (Figure 2A,B). These results suggest that incomplete reprogramming of H3K9me3 occurs during the early development of porcine SCNT embryos.

### 2.2. DNA Methylation Levels in IVF and SCNT Embryos

We investigated the expression levels of *DNMT1*, *DNMT3a*, and *DNMT3b* in porcine IVF and SCNT embryos. Interestingly, the expression levels of DNMTs decreased from the pronuclear to the blastocyst stage in both IVF and SCNT embryos (Figure S1C–E). In addition, the expression levels of DNMTs were significantly higher in SCNT embryos than in IVF embryos at all stages (Figure 2C–E). These results suggest that aberrant DNA methylation occurs during the early development of porcine SCNT embryos.

### 2.3. Effects of Chaetocin on the Developmental Competence of Porcine SCNT Embryos

**Figure 3:**
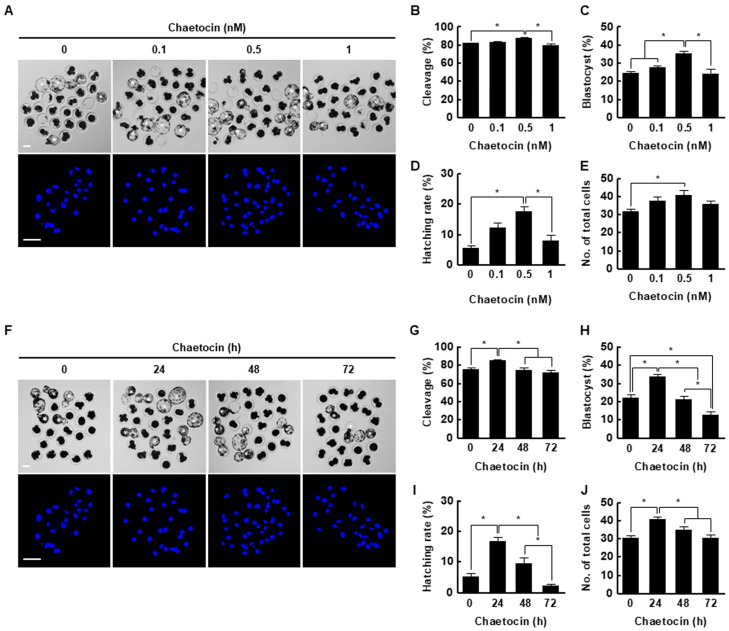
Effect of chaetocin on in vitro development of porcine SCNT embryos. (**A**) Representative bright-field (upper, bar = 50 µm) and nuclear-stained (lower, bar = 100 µm) images of blastocysts cultured in the presence of chaetocin for 24 h. Quantification of the (**B**) cleavage rate, (**C**) blastocyst formation rate, (**D**) hatching rate, and (**E**) total cell number (0; *n* = 148, 0.1; *n* = 149, 0.5; *n* = 148, 1; *n* = 149). (**F**) Representative bright-field (upper, bar = 50 µm) and nuclear-stained (lower, bar = 100 µm) images of blastocysts treated with 0.5 nM chaetocin. Quantification of the (**G**) cleavage rate, (**H**) blastocyst formation rate, (**I**) hatching rate, and (**J**) total cell number following treatment with 0.5 nM chaetocin (0; *n* = 132, 24; *n* = 132, 48; *n* = 132, 72; *n* = 132). The data are from four independent experiments and are means ± SEM (* *p* < 0.05).

We treated porcine SCNT embryos with 0, 0.1, 0.5, or 1 nM chaetocin for 24 h after activation. The cleavage rate, blastocyst formation rate, hatching rate, and total cell number of porcine SCNT embryos were significantly increased by 0.5 nM chaetocin (Figure 3A–E, Table S2). Next, we investigated the effects of the duration (0, 24, 48, or 72 h) of treatment with 0.5 nM chaetocin. Treatment with chaetocin for 24 h significantly increased the developmental competence of SCNT embryos compared to the control (Figure 3F–J, Table S3). Therefore, we applied 0.5 nM chaetocin for 24 h in subsequent experiments.

**Figure 4:**
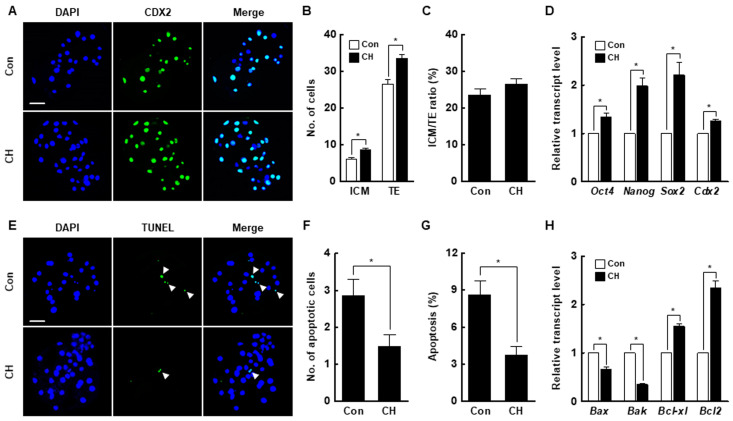
Effect of chaetocin on the developmental competence of porcine SCNT embryos. (**A**) Representative immunofluorescence images of Cdx2/DAPI in blastocysts. Embryos were stained for CDX2 (green) and DNA (DAPI, blue). Bar = 50 µm. Quantification of the (**B**) inner cell mass (ICM) and trophectoderm (TE) cell numbers, and (**C**) ICM/TE ratios (*n* = 20 per group). (**D**) Quantitative real-time polymerase chain reaction (qRT-PCR) results for pluripotency-related genes in blastocysts (*n* = 3 per group). (**E**) Terminal deoxynucleotidyl transferase-mediated 2′-deoxyuridine-5′-triphosphate (dUTP)-digoxigenin nick end-labeling (TUNEL) assay of blastocysts. Embryos were stained for TUNEL (green, white arrow) and DNA (DAPI, blue). Bar = 50 µm. Quantification of the (**F**) number and (**G**) proportion of apoptotic cells (*n* = 21 per group). (**H**) qRT-PCR results for apoptosis-related genes in blastocysts (*n* = 3 per group). The data are from three independent experiments and are means ± SEM (* *p* < 0.05).

Chaetocin markedly increased the inner cell mass (ICM) and trophectoderm (TE) cell numbers of SCNT embryos compared to the control but did not affect the ICM to TE cell ratio (Figure 4A–C, Table S4). The expression levels of ICM (octamer-binding transcription factor 4 [*Oct4*], nanog homeobox [*Nanog*], and SRY-box transcription factor 2 [*Sox2*])- and TE (caudal type homeobox 2; *Cdx2*)-related genes were considerably increased by chaetocin (Figure 4D). Moreover, chaetocin significantly reduced the rate of apoptosis, the number of apoptotic cells, and the expression levels of pro-apoptosis genes (BCL2 associated X [*Bax*] and Bcl-2 homologous antagonist killer [*Bak*]) and increased the expression levels of anti-apoptosis genes (B-cell lymphoma [*Bcl*]*-xl* and *Bcl2*) compared to the control (Figure 4E–H, Table S5). Therefore, chaetocin enhances the developmental competence of porcine SCNT embryos.

### 2.4. Effects of Chaetocin on H3K9me3 and Global DNA Methylation During Porcine SCNT Embryo Development

**Figure 5:**
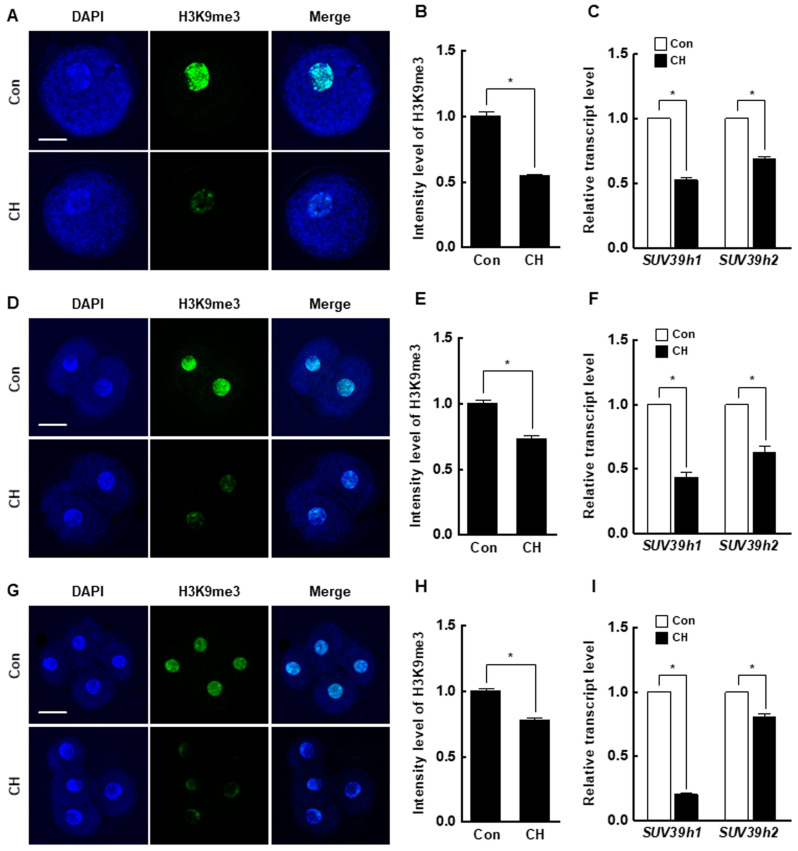
Effect of chaetocin on the H3K9me3 level of porcine SCNT embryos. (**A**) Representative immunofluorescence images of H3K9me3 in SCNT embryos at the pronuclear stage. Embryos were stained for H3K9me3 (green) and DNA (DAPI, blue). Bar = 50 µm. (**B**) Quantification of fluorescence intensity at the pronuclear stage (*n* = 20 per group). (**C**) qRT-PCR results for H3K9me3-specific methyltransferases (*suv39h1*, *suv39h2*) at the pronuclear stage (*n* = 3 per group). (**D**) Representative immunofluorescence images of H3K9me3 in SCNT embryos at the two-cell stage. Embryos were stained for H3K9me3 (green) and DNA (DAPI, blue). Bar = 50 µm. (**E**) Quantification of the fluorescence intensity at the two-cell stage (*n* = 18 per group). (**F**) qRT-PCR results for H3K9me3-specific methyltransferases (*suv39h1*, *suv39h2*) at the two-cell stage (*n* = 3 per group). (**G**) Representative immunofluorescence images of H3K9me3 in SCNT embryos at the four-cell stage. Embryos were stained for H3K9me3 (green) and DNA (DAPI, blue). Bar = 50 µm. (**H**) Quantification of the fluorescence intensity at the four-cell stage (*n* = 18 per group). (**I**) qRT-PCR results for H3K9me3-specific methyltransferases (*suv39h1*, *suv39h2*) at the four-cell stage (*n* = 3 per group). The data are from three independent experiments and are means ± SEM (* *p* < 0.05).

**Figure 6:**
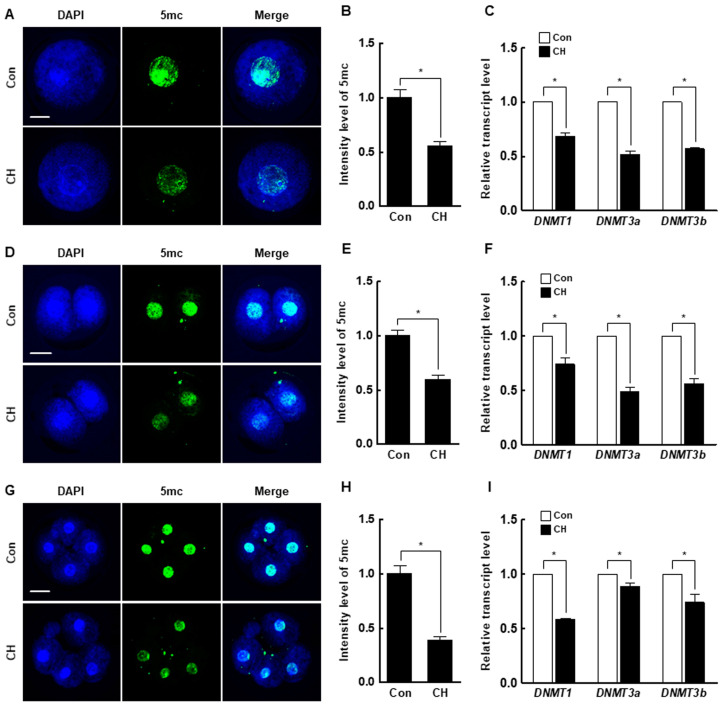
Effect of chaetocin on global DNA methylation of porcine SCNT embryos. (**A**) Representative immunofluorescence images of 5-mc in SCNT embryos at the pronuclear stage. Embryos were stained for 5-mc (green) and DNA (DAPI, blue). Bar = 50 µm. (**B**) Quantification of fluorescence intensity at the pronuclear stage (*n* = 20 per group). (**C**) qRT-PCR results for DNMTs (*DNMT1*, *DNMT3a*, *DNMT3b*) at the pronuclear stage (*n* = 3 per group). (**D**) Representative immunofluorescence images of 5-mc in SCNT embryos at the two-cell stage. Embryos were stained for 5-mc (green) and DNA (DAPI, blue). Bar = 50 µm. (**E**) Quantification of fluorescence intensity at the two-cell stage (*n* = 20 per group). (**F**) qRT-PCR results for DNMTs (*DNMT1*, *DNMT3a*, *DNMT3b*) at the two-cell stage (*n* = 3 per group). (**G**) Representative immunofluorescence images of 5-mc in SCNT embryos at the four-cell stage. Embryos were stained for 5-mc (green) and DNA (DAPI, blue). Bar = 50 µm. (**H**) Quantification of fluorescence intensity at the four-cell stage (*n* = 20 per group). (**I**) qRT-PCR results for DNMTs (*DNMT1*, *DNMT3a*, *DNMT3b*) at the four-cell stage (*n* = 3 per group). The data are from three independent experiments and are means ± SEM (* *p* < 0.05).

At the pronuclear stage, the H3K9me3 level was significantly decreased by chaetocin compared to the control (Figure 5A,B), and the expression levels of *suv39h1* and *suv39h2* were also considerably decreased by chaetocin (Figure 5C). The results for two- and four-cell stage embryos were similar to those for pronuclear stage embryos (Figure 5D–I). Interestingly, the 5-methylcytosine (5-mc) levels and expression level of DNMTs (*DNMT1*, *DNMT3a*, and *DNMT3b*) were significantly decreased by chaetocin treatment at the pronuclear, two-, and four-cell stages of SCNT embryos (Figure 6). Therefore, chaetocin restores aberrant epigenetic reprogramming by regulating H3K9me3 and global DNA methylation during the development of porcine SCNT embryos.

### 2.5. Effects of Chaetocin on Autophagic Activity and Maternal mRNA Levels in Porcine SCNT Embryos

**Figure 7:**
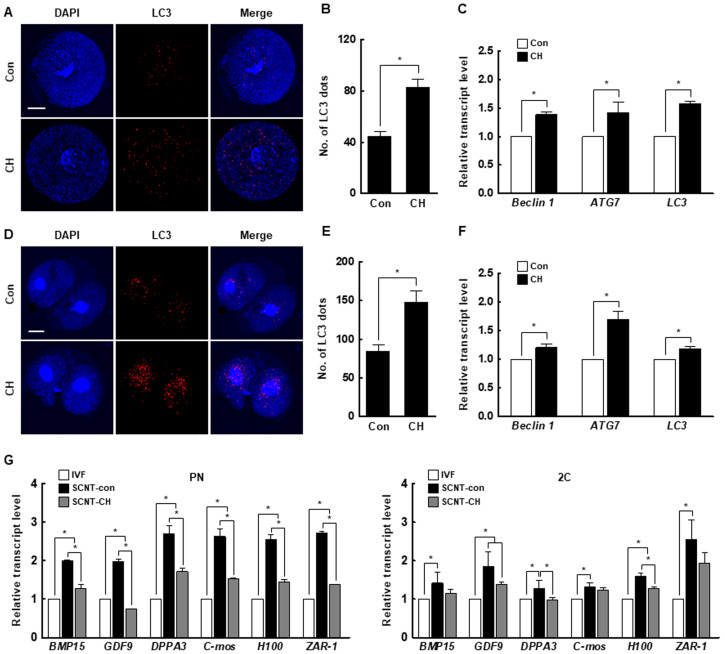
Effect of chaetocin on the autophagic activity and maternal mRNA level of porcine SCNT embryos. (**A**) Representative immunofluorescence images of microtubule-associated protein 1A/1B-light chain 3 (LC3) at the pronuclear stage. Embryos were stained for LC3 (red) and DNA (DAPI, blue). Bar = 50 µm. (**B**) Quantification of the fluorescence intensity at the pronuclear stage. (*n* = 21 per group) (**C**) qRT-PCR results for autophagy-related genes at the pronuclear stage (*n* = 3 per group). (**D**) Representative immunofluorescence images of LC3 in SCNT embryos at the two-cell stage. Embryos were stained for LC3 (red) and DNA (DAPI, blue). Bar = 50 µm. (**E**) Quantification of the fluorescence intensity at the two-cell stage. (*n* = 20 per group) (**F**) qRT-PCR results for autophagy-related genes at the two-cell stage (*n* = 3 per group). (**G**) qRT-PCR results for maternal factor-related genes at the pronuclear (left) and two-cell (right) stages (*n* = 3 per group). The data are from three independent experiments and are means ± SEM (* *p* < 0.05).

We investigated the number of microtubule-associated protein 1A/1B-light chain 3 (LC3) dots, a marker of autophagy induction, and the expression levels of autophagy-related genes in porcine SCNT embryos by immunofluorescence and qRT-PCR. At the pronuclear and two-cell stages, both were significantly increased by chaetocin (Figure 7A–F). In addition, the expression levels of the maternal-related genes (bone morphogenetic protein 15 [*BMP15*], growth differentiation factor-9 [*GDF9*], developmental pluripotency-associated protein 3 [*DPPA3*], MOS proto-oncogene, serine/threonine kinase [*C-mos*], oocyte histone H1 linker [*H100*], and zygote arrest 1 [*ZAR-1*]) were significantly higher in control than in IVF embryos. The expression levels of most of these genes in pronuclear- and two-cell stage SCNT embryos were decreased by chaetocin (Figure 7G). Therefore, chaetocin enhances the developmental competence of porcine SCNT embryos by inducing autophagic activity to remove obsolete maternal factors.

## 3. Discussion

Transgenic pigs are used in biomedical and regenerative medicine research as disease and xenotransplantation models [26,27]. To efficiently produce transgenic pigs, it is important to produce SCNT embryos with high developmental competence. However, the developmental competence of SCNT embryos is low, possibly as a result of developmental defects caused by incomplete reprogramming of the genome of the somatic donor cell [28,29]. Epigenetic modifications, including DNA methylation and histone changes, are implicated in nuclear reprogramming and chromatin organization, DNA accessibility, and gene expression [30]. Histone methylation is associated with transcriptional repression or activation and plays a crucial role in the development of in vivo- or in vitro-fertilized mammalian embryos [31]. SCNT embryos reportedly harbor aberrant levels of H3K9me3, *suv39h1*, and *suv39h2* transcripts compared to IVF embryos, resulting in aberrant reprogramming and defective embryonic development [8,9,10,12,32]. In this study, the H3K9me3 level was significantly higher in SCNT embryos than in IVF embryos at the pronuclear, two-cell, and four-cell stages. SCNT embryos also showed higher levels of *suv39h1* and *suv39h2* than IVF embryos. Therefore, we attempted to enhance the developmental competence of porcine SCNT embryos using chaetocin, an inhibitor of *suv39h1* and *suv39h2*.

Microinjection of the mRNAs of Kdm4d, Kdm4a, and Kdm4e H3K9-specific demethylases, into mouse, human, and bovine SCNT embryos significantly decreases the H3K9me3 level and increases developmental efficiency [8,9,33]. In the previous study, chaetocin was used to downregulate the H3K9me3 level in SCNT embryos to prevent damage following microinjection of Kdm4 mRNA; however, 10 nM chaetocin did not improve the developmental rate of ovine SCNT embryos [32]. In another study, treatment of porcine SCNT embryos at the four-cell stage, but not the one- or two-cell stage, with 10 nM chaetocin for 6 h significantly increased the rate of development [34]. In our study, we found an aberrant H3K9me3 level in the pronuclear, two-cell, and four-cell stage of SCNT embryos compared to IVF embryos. Therefore, it is important to correct aberrant reprogramming beginning at the pronuclear stage of SCNT embryos. Moreover, SCNT embryos are typically transferred to the recipient oviduct as early as the one- or two-cell stage because of the reduced efficiency of in vitro culture conditions compared to in vivo [35,36]. For these reasons, the application of histone and DNA methyltransferase inhibitors, such as BIX-01294, MM-102, RG108, DZNep, and UNC0642, at the early stages after activation accelerates the in vitro development of SCNT embryos [37,38,39,40]. In this study, treatment with 0.5 nM chaetocin for 24 h after activation significantly enhanced the developmental competence of porcine SCNT embryos in terms of the cleavage rate, blastocyst formation rate, hatching rate, total cell number, and cell survival rate. Moreover, the expression levels of *oct4*, *nanog*, and *sox2* were markedly increased. This is consistent with previous reports that H3K9me3 marks heterochromatin foci in the ICM and that reducing the H3K9me3 level upregulates the expression of pluripotency-related genes [39,41,42,43].

Chaetocin is the first inhibitor of histone lysine methyltransferase, which was found to be a specific inhibitor of the SUV39 family, such as *suv39h1*, *suv39h2*, and *G9a* [20,44]. Previous studies reported that chaetocin could reduce *suv39h1*, *suv39h2*, and *G9a* mRNA or their protein in ovine cells and human cancer cells [22,32,45,46]. In the current study, we also showed that chaetocin significantly reduced not only the H3K9me3 levels but also the expression of *suv39h1* and *suv39h2* in the early stage of porcine SCNT embryos, indicating that chaetocin could efficiently downregulate the H3K9me3 levels possibly via reduction of the expression or protein stability of H3K9 methyltransferases. However, the exact mechanism of chaetocin governing the reduction of H3K9me3 has not yet been fully elucidated or was largely unknown until recently. Thus, the understanding of chaetocin function as a transcriptional modifier of *suv39h1* and *suv39h2* genes requires further detailed investigation, such as ChiP-seq analysis between modification types of histone H3 and the promoter region of both genes.

Global DNA methylation, which occurs on cytosine-guanine dinucleotides, is important for epigenetic reprogramming and normal embryonic development in mammals [47]. DNA methylation is regulated by two enzyme systems, namely, ten-eleven translocation methylcytosine dioxygenase (TET) family and DNMTs [48,49]. Inhibition or the absence of DNMTs causes passive DNA demethylation, whereas TET proteins are involved in the oxidation of 5-mc and promote locus-specific reversal of DNA methylation to promote demethylation (active DNA demethylation). In addition, the importance of TET-mediated demethylation has been elucidated by investigating the expression of imprinting-related genes of paternal chromosomes during reprogramming [50], although TET-mediated active DNA demethylation was not examined in the current study. *DNMT1* is the most abundant one and acts to maintain methylation, whereas *DNMT3a* and *DNMT3b* are responsible for de novo methylation [31]. SCNT embryos showed abnormal DNA methylation due to the use of highly methylated somatic cells, which reduces developmental competence [7,51]. A previous study reported that *suv39h1*- and *suv39h2*-knockout embryonic stem cells show decreased DNA methylation [16], and *suv39h1* and *suv39h2* directly interact with HP1 to methylate heterochromatin regions [17], indicating that DNMTs have functional interactions with H3K9me3 methyltransferases. Furthermore, DNMTs regulate *suv39h1* and *suv39h2*, suggesting that H3K9me3 and DNA methylation act in concert to maintain a repressed chromatin state. In this study, the DNMTs’ expression levels in SCNT embryos were higher than in IVF embryos and were significantly restored by chaetocin. Moreover, chaetocin treatment noticeably downregulated the 5-mc levels, an indicator of DNA methylation, at the early stage of SCNT embryos. These results demonstrate that chaetocin enhances epigenetic reprogramming in porcine SCNT embryos by reducing the levels of H3K9me3 and global DNA methylation.

Autophagy is a crucial cellular mechanism that degrades unnecessary or dysfunctional cellular components to maintain intracellular homeostasis [52]. During the oocyte-to-embryo transition, unnecessary maternal mRNAs and proteins in oocytes are degraded and new proteins are synthesized; autophagy plays an important role in these processes [53,54]. Thus, autophagic activity is considered important for embryonic development. In addition, autophagy is implicated in the modulation of somatic cell reprogramming, such as the homeostasis of pluripotency-related proteins [55]. Indeed, rapamycin, an activator of autophagy, reportedly increases the reprogramming efficiency of induced pluripotent stem cells [56]. In two previous studies on SCNT embryos, rapamycin restored reprogramming efficiency and embryonic development [54,57]. In the present study, the number of LC3 dots and the expression levels of autophagy-related genes were increased by chaetocin, indicating that chaetocin induces autophagic activity in porcine SCNT embryos. These results are consistent with previous reports that chaetocin induces autophagic activity by increasing the LC3 level in cancer cells [58,59]. Moreover, the expression level of most maternal-related genes was higher in SCNT than in IVF embryos but was decreased by chaetocin. These results strongly suggest that chaetocin treatment in early stage of SCNT embryos enhances epigenetic reprogramming and developmental competence by inducing autophagic activity, and improving the efficiency of degradation of maternal mRNA in pigs.

In conclusion, an aberrant H3K9me3 level is a major epigenetic barrier during the development of porcine SCNT embryos. We found that chaetocin treatment of early stage SCNT embryos increased developmental competence by enhancing epigenetic reprogramming and autophagic activity. Therefore, chaetocin could be applied to enhance our understanding of the role of H3K9me3 in the regulation of autophagy in early stage SCNT embryos. Our findings will enable the production of SCNT embryos with high developmental competence and the generation of transgenic pigs for biomedical research.

## 4. Materials and Methods

### 4.1. Ethics Statement

This study was carried out in strict accordance with the recommendations of the Korea Research Institute of Bioscience and Biotechnology (KRIBB) Institutional Animal Care and Use Committee (Approval No. KRIBB-AEC-19118, 22/04/2019).

### 4.2. Chemicals

All chemicals and reagents were purchased from Sigma-Aldrich Chemical Co. (St. Louis, MO, USA) unless otherwise indicated.

### 4.3. Oocyte Collection and In Vitro Maturation (IVM)

To obtain porcine oocytes, ovaries were collected from prepubertal gilts at a nearby local slaughterhouse and transported to the laboratory in 0.9% saline containing 75 µg/mL potassium penicillin G and 50 µg/mL streptomycin sulfate at 38.5 °C within 2 h. Cumulus–oocyte complexes (COCs) were aspirated from follicles (3–6 mm in diameter) using an 18-gauge needle into a disposable 10 mL syringe. Collected COCs were washed three times in Tyrode’s Albumin Lactate Pyruvate-HEPES medium and then approximately 50 COCs were sequentially matured in 500 µL of IVM medium in a 4-well multi-dish (Nunc, Roskilde, Denmark) for 44 h at 38.5 °C in 5% CO_2_ in air. The IVM medium consisted of tissue culture medium 199 supplemented with 10% porcine follicular fluid, 0.57 mM cysteine, 10 ng/mL β-mercaptoethanol, 10 ng/mL epidermal growth factor, 10 IU/mL pregnant mare serum gonadotropin, and 10 IU/mL human chorionic gonadotropin. After 22 h of IVM, COCs were further cultured in IVM medium without hormones for another 22 h. After 44 h of IVM, expanded cumulus cells with oocytes were treated with 0.1% hyaluronidase and then removed after vortexing for 1 min. Matured MII oocytes with a visible polar body, regular morphology, and homogenous cytoplasm were used for experiments.

### 4.4. In Vitro Fertilization (IVF) and In Vitro Culture (IVC)

IVF was performed in a modified Tris-buffered medium (mTBM), consisting of 113.1 mM NaCl, 3 mM KCl, 7.5 mM CaCl_2_·2H_2_O, 20 mM Tris (Fisher Scientific, Fair Lawn, NJ, USA), 11 mM glucose, and 5 mM sodium pyruvate, and no antibiotics. MII oocytes were washed three times in mTBM containing 2.5 mM caffeine sodium benzoate and 1 mg/mL bovine serum albumin (BSA), and 10–15 oocytes were placed into a 48 µL droplet of IVF medium under mineral oil pre-equilibrated at 38.5 °C in 5% CO_2_ in air. For preparation of the spermatozoa using the swim-up method prior to fertilization, freshly ejaculated semen was washed three times with sperm washing medium (Dulbecco’s phosphate-buffered saline [DPBS; Gibco-BRL, Grand Island, NY, USA]) supplemented with 1 mg/mL BSA, 100 µg/mL penicillin G, and 75 µg/mL streptomycin sulfate). After washing, 2 mL of sperm washing medium were gently added to the spermatozoa pellet and incubated for 15 min at 38.5 °C in 5% CO_2_ in air. After incubation, supernatant was washed with mTBM, and resuspended with 1 mL mTBM. Then, 2 µL of diluted spermatozoa were added to 48 µL of mTBM containing 10–15 oocytes to a final concentration of 1.5 × 10^5^ spermatozoa/mL. Oocytes were co-incubated with the spermatozoa for 6 h at 38.5 °C in 5% CO_2_ in air. After 6 h, oocytes were stripped by gentle pipetting and transferred to IVC medium, consisting of porcine zygote medium-3 (PZM-3) containing 4 mg/mL BSA, for culture at 38.5 °C in 5% CO_2_ in air. PZM-3 consisted of 108 mM NaCl, 10 mM KCl, 0.4 mM MgSO_4_·7H_2_O, 0.35 mM KH_2_PO_4_, 25.07 mM NaHCO_3_, 0.2 mM sodium pyruvate, 2 mM Ca-lactate·5H_2_O, and 50 μg/mL gentamicin sulfate.

### 4.5. Primary Cell Establishment and Donor Cell Preparation

Porcine kidney was obtained from a neonatal pig by surgical operation. Harvested kidney tissues were stored in DPBS washing buffer containing 10% (v/v) penicillin/streptomycin (Invitrogen, Carlsbad, CA, USA) on ice until isolation. Kidney biopsies (2 × 1 × 1 cm) were washed three times in washing buffer, chopped (0.3 × 0.3 × 0.3 cm), and washed with Dulbecco’s modified eagle’s medium (DMEM; Invitrogen). Small pieces of kidney tissue were placed in 60 mm culture dishes and cultured in DMEM containing 10% fetal bovine serum (FBS; Gibco), 10 ng/mL basic fibroblast growth factors (R&D SYSTEM, Minneapolis, MN, USA), and 1% (v/v) penicillin/streptomycin at 38.5 °C in 5% CO_2_ in air until confluent. Donor cells were used at passages 4 to 6 for SCNT. To synchronize the cell cycle at the G0–G1 phase, kidney cells were cultured after reaching confluency and then further cultured in culture medium containing 0.5% FBS for 3 days. Donor cells for SCNT were washed with DPBS and digested with 0.25% trypsin-EDTA for 3 min, then trypsin activity was blocked with DMEM containing 10% FBS. The cells were spun down at a low speed (150× *g*) for 2 min and resuspended with DPBS.

### 4.6. Somatic Cell Nuclear Transfer (SCNT) and Chaetocin Treatment

SCNT were performed as previously described [60]. MII oocytes in PB1 medium (DPBS supplemented with 4 mg/mL BSA, 75 µg/mL penicillin G, and 50 µg/mL streptomycin sulfate) containing 7.5 µg/mL cytochalasin B were cut using a sharp pipette, and then the first polar body and cytoplasm-containing chromosomes at metaphase II were removed using the squeezing method under an inverted microscope (DMI 3000B; LEICA, Wetzlar, Germany) equipped with a micromanipulator (NT-88-V3; Nikon Narishige, Tokyo, Japan). Porcine kidney cells were suitable for the donor cell resource to produce SCNT embryos, as they showed higher proliferation and blastocyst formation rates after SCNT compared to porcine fetal or ear fibroblast cells [61]. Donor cells were selected with good refractivity and placed into the perivitelline space. A single cell–oocyte couplet was placed between two parallel electrodes (CUY 5100-100; Nepa gene, Ichikawa, Japan) and activated by one direct current pulse of 0.24 kV/cm for 50 µs using an Electro Cell Fusion generator in fusion medium consisting of 280 mM mannitol containing 0.1 mM CaCl_2_·2H_2_O, 0.2 mM MgSO_4_·7H_2_O, and 0.01% polyvinyl alcohol (PVA), and incubated at 38.5 °C in 5% CO_2_ in air. After 2 h, oocyte–cell couplets that were completely fused as observed under an inverted microscope were selected and activated by one direct current pulse of 1.2 kV/cm for 50 µs in activation medium consisting of 280 mM mannitol containing 0.1 mM CaCl_2_·2H_2_O, 0.2 mM MgSO_4_·7H_2_O, 0.01% PVA, and 0.5 mM HEPES, and then cultured in post-activation medium, which consisted of IVC medium supplemented with 5 µg/mL cytochalasin B and 2mM 6-dimethylaminopurine, for 4 h at 38.5 °C in 5% CO_2_ in air. After activation, the activated embryos were transferred to IVC medium at 38.5 °C in 5% CO_2_ in air.

To confirm the optimal conditions for chaetocin treatment during porcine SCNT embryo development, activated embryos were cultured in activation medium with various concentrations of chaetocin (0, 0.1, 0.5, and 1 nM) for 24 h after activation. The concentration that caused the highest percentage of embryos to develop (0.5 nM) was used for various durations of treatment (0, 24, 48, and 72 h) after activation. The cleavage and blastocyst rates were determined at 48 and 144 h, respectively.

### 4.7. Indirect Immunofluorescence

Embryos of every stage derived from IVF and SCNT were washed in DPBS supplemented with 0.1% PVA (DPBS-PVA) for 10 min each. For membrane permeabilization, the fixed embryos were incubated in DPBS containing 0.5% Triton X-100 for 40 min at room temperature (RT). For the staining of global methylation, permeabilized embryos were additionally stored in 1 M HCl for 30 min at 38.5 °C. Subsequently, embryos were washed three times in DPBS-PVA and stored in blocking medium, which consisted of DPBS containing 4mg/mL BSA for 1 h at RT. The embryos were incubated with primary antibodies H3K9me3 (1:1000 dilution, Abcam, Cambridge, MA, USA), 5-mc (1:200 dilution, Calbiochem, San Diego, CA, USA), or LC3 (1:100 dilution; #2775, Cell Signaling Technology, Danvers, MA, USA) overnight at 4 °C. After washing three times with DPBS containing 0.05% Tween 20, the embryos were incubated with the secondary antibody, Alexa Fluor 488 goat anti rabbit or mouse IgG (1:1000 for H3K9me3 or 1:200 for 5-mc and LC3) for 1 h at RT. After washing three times with DPBS containing 0.05% Tween 20, embryos were mounted on clean glass slides with 4′,6′-diamidino-2-phenylindole (DAPI) and observed under a fluorescence microscope (Olympus, Tokyo, Japan). Approximately five to eight embryos were used in the immunocytochemistry in each independent experiment.

### 4.8. qRT-PCR

Poly(A) mRNAs were extracted from embryos using the Dynabeads mRNA Direct kit (Invitrogen) according to the manufacturer’s protocol. Briefly, after thawing, samples were lysed in 200 µL of lysis/binding buffer at RT for 10 min, and 20 µL of Dynabeads oligo(dT)_25_ were added to each sample. The beads were hybridized for 5 min and then separated from the binding buffer using a Dynal magnetic bar (Invitrogen). Bound poly(A) mRNAs and beads were washed with buffers A and B and then separated by adding 10 µL of Tris buffer. The resulting poly(A) mRNAs were reverse transcribed in 20-µL reactions containing oligo(dT)_20_, 5× RT buffer (containing 25 mM Mg^2+^), 10 U of the RNase inhibitor ReverTra Ace (Toyobo, Osaka, Japan), and a 10 mM mixture of dNTPs. The secondary RNA structure was denatured by incubating at 42 °C for 60 min to facilitate cDNA production. The reaction was terminated by incubation at 99 °C for 5 min. The resulting cDNA was used as a template for PCR amplification. The following PCR conditions were used: 95 °C for 30 s, 60°C for 30 s, and 72 °C for 30 s, followed by extension at 72 °C for 5 min. The Mx3000P QPCR system (Agilent, Santa Clara, CA, USA) and SYBR premix Ex Taq (Takara Bio Inc, Shiga, Japan) were used for qRT-PCR. The threshold cycle (Ct) is defined as the fractional cycle number at which the fluorescence passes a fixed threshold above baseline. For the comparative analyses, mRNA expression levels were normalized to glyceraldehyde-3-phosphate dehydrogenase (GAPDH) and are expressed as the fold change. The sample delta Ct (^SΔCT^) value was calculated from the difference between the Ct values of GAPDH and the target genes. The relative gene expression levels between the samples and the controls were determined using the formula 2^−(SΔCT−CΔCT)^. The primers used in the current study are listed in Table S1.

### 4.9. CDX2 Staining

Blastocysts were fixed in 4% paraformaldehyde overnight at 4 °C and washed three times in DPBS-PVA for 10 min each. For membrane permeabilization, the fixed blastocysts were incubated in PBS containing 0.5% Triton X-100 for 40 min at RT. Subsequently, blastocysts were washed three times in DPBS-PVA and stored in DPBS-PVA supplemented with 1 mg/mL BSA (DPBS-PVA-BSA) at 4°C overnight. The blastocysts were blocked with 10% normal goat serum for 45 min and then incubated overnight at 4 °C with primary antibody, mouse monoclonal anti-Cdx2 (an undiluted solution; Biogenex Laboratories Inc., San Ramon, CA, USA). Subsequently, the blastocysts were washed three times in DPBS-PVA-BSA for 10 min each and incubated for 40 min at RT with conjugated secondary antibodies, Alexa-Fluor-488-labeled goat anti-mouse IgG (1:200 in DPBS-PVA-BSA). After the blastocysts were washed three times in DPBS-PVA-BSA for 10 min each, the DNA was stained with 2 µg/mL DAPI. DAPI-labeled or Cdx2-positive nuclei were observed using a fluorescence microscope (Olympus). Approximately seven to eight blastocysts per treatment group were used in the immunocytochemistry in each independent experiment.

### 4.10. Terminal Deoxynucleotidyl Transferase-Mediated dUTP-Digoxygenin Nick End-Labeling Assay (TUNEL)

To evaluate apoptotic blastomeres in blastocysts, a TUNEL assay was performed using an in situ cell death detection kit (Roche, Basel, Switzerland). Blastocysts were washed three times in DPBS-PVA and fixed in 4% paraformaldehyde overnight at 4 °C. Fixed blastocysts were permeabilized in DPBS containing 0.5% Triton X-100 at RT for 60 min. Nonspecific binding sites were blocked by incubation with DPBS containing 10 mg/mL BSA for 1 h. Subsequently, blastocysts were washed three times with DPBS-PVA and stained with fluorescein-conjugated dUTP and terminal deoxynucleotidyl transferase for 1 h at 38.5 °C. Subsequently, the blastocysts were washed three times with DPBS-PVA and mounted on clean glass slides with DAPI. DAPI-labeled or TUNEL-positive nuclei were observed under a fluorescence microscope (Olympus). Total and apoptotic cell numbers per blastocyst were judged by counting the nuclei with blue (DAPI) and green (TUNEL) signals. Approximately seven to eight blastocysts per treatment group were used in the TUNEL assays in each independent experiment.

### 4.11. Statistical Analyses

All experiments were repeated at least three times. Data are expressed as the means ± standard error of the mean (SEM). Data were analyzed by analysis of variance (ANOVA), followed by Tukey’s multiple range test (Figure 3; data on development of SCNT embryos, Figure 7; maternal factor-related genes qRT-PCR) or Student’s t-test (Figure 1; data on H3K9me3 levels, Figure 2; data on H3K9me3- or DNMT-related genes qRT-PCR, Figure 4; data on CDX2, TUNEL, and pluripotency- or apoptosis-related genes qRT-PCR, Figure 5; data on H3K9me3 levels and H3K9me3-related genes qRT-PCR, Figure 6; data on 5-mc levels and DNMT-related genes qRT-PCR, Figure 7; data on LC3 and autophagy-related genes qRT-PCR), using SigmaStat Software (SPSS Inc., Chicago, IL, USA). *p*-values less than 0.05 were considered to indicate statistical significance.

## Supplementary Materials

Supplementary materials can be found at https://www.mdpi.com/1422-0067/21/14/4836/s1.

## Abbreviations

SCNT	somatic cell nuclear transfer
H3K9me3	histone H3 lysine 9 trimethylation
KDM	H3 lysine9 specific demethylase
DNMTs	DNA methyltransferases
HP1	heterochromatin protein 1
ICM	inner cell mass
TE	trophectoderm
IVM	in vitro maturation
COCs	Cumulus–oocyte complexes
IVF	in vitro fertilization
IVC	in vitro culture
mTBM	modified Tris-buffered medium
BSA	bovine serum albumin
DPBS	Dulbecco’s phosphate-buffered saline
DMEM	Dulbecco’s Modified Eagle’s Medium
FBS	fetal bovine serum
PVA	polyvinyl alcohol
DPBS-PVA	DPBS supplemented with 0.1% PVA
RT	room temperature
5-mc	5-methylcytosine
DAPI	4′,6′-diamidino-2-phenylindole
qRT-PCR	Quantitative real-time polymerase chain reaction
GAPDH	glyceraldehyde-3-phosphate dehydrogenase
DPBS-PVA-BSA	DPBS-PVA supplemented with 1 mg/mL BSA
TUNEL	terminal deoxynucleotidyl transferase-mediated dUTP-digoxigenin nick end-labeling
SEM	standard error of the mean
LC3	microtubule-associated protein 1A/1B-light chain 3
TET	ten-eleven translocation methylcytosine dioxygenase
PZM-3	porcine zygote medium-3
ANOVA	analysis of variance

## References

[1] Simon G.A., Maibach H.I. (2000). The pig as an experimental animal model of percutaneous permeation in man: Qualitative and quantitative observations—An overview. Skin Pharmacol. Appl. Skin Physiol..

[2] Prather R.S., Hawley R.J., Carter D.B., Lai L., Greenstein J.L. (2003). Transgenic swine for biomedicine and agriculture. Theriogenology.

[3] Whitelaw C.B., Sheets T.P., Lillico S.G., Telugu B.P. (2016). Engineering large animal models of human disease. J. Pathol..

[4] Song Y., Hai T., Wang Y., Guo R., Li W., Wang L., Zhou Q. (2014). Epigenetic reprogramming, gene expression and in vitro development of porcine SCNT embryos are significantly improved by a histone deacetylase inhibitor--m-carboxycinnamic acid bishydroxamide (CBHA). Protein Cell.

[5] Wilmut I., Schnieke A.E., McWhir J., Kind A.J., Campbell K.H. (1997). Viable offspring derived from fetal and adult mammalian cells. Nature.

[6] Wakayama T., Perry A.C., Zuccotti M., Johnson K.R., Yanagimachi R. (1998). Full-term development of mice from enucleated oocytes injected with cumulus cell nuclei. Nature.

[7] Peat J.R., Reik W. (2012). Incomplete methylation reprogramming in SCNT embryos. Nat. Genet..

[8] Matoba S., Liu Y., Lu F., Iwabuchi K.A., Shen L., Inoue A., Zhang Y. (2014). Embryonic development following somatic cell nuclear transfer impeded by persisting histone methylation. Cell.

[9] Chung Y.G., Matoba S., Liu Y., Eum J.H., Lu F., Jiang W., Lee J.E., Sepilian V., Cha K.Y., Lee D.R. (2015). Histone Demethylase Expression Enhances Human Somatic Cell Nuclear Transfer Efficiency and Promotes Derivation of Pluripotent Stem Cells. Cell Stem Cell.

[10] Cao Z., Li Y., Chen Z., Wang H., Zhang M., Zhou N., Wu R., Ling Y., Fang F., Li N. (2015). Genome-Wide Dynamic Profiling of Histone Methylation during Nuclear Transfer-Mediated Porcine Somatic Cell Reprogramming. PLoS ONE.

[11] Zhai Y., Li W., Zhang Z., Cao Y., Wang Z., Zhang S., Li Z. (2018). Epigenetic states of donor cells significantly affect the development of somatic cell nuclear transfer (SCNT) embryos in pigs. Mol. Reprod. Dev..

[12] Liu Z., Cai Y., Wang Y., Nie Y., Zhang C., Xu Y., Zhang X., Lu Y., Wang Z., Poo M. (2018). Cloning of Macaque Monkeys by Somatic Cell Nuclear Transfer. Cell.

[13] Wang C., Liu X., Gao Y., Yang L., Li C., Liu W., Chen C., Kou X., Zhao Y., Chen J. (2018). Reprogramming of H3K9me3-dependent heterochromatin during mammalian embryo development. Nat. Cell Biol..

[14] Peters A.H., O’Carroll D., Scherthan H., Mechtler K., Sauer S., Schofer C., Weipoltshammer K., Pagani M., Lachner M., Kohlmaier A. (2001). Loss of the Suv39h histone methyltransferases impairs mammalian heterochromatin and genome stability. Cell.

[15] Ruan D., Peng J., Wang X., Ouyang Z., Zou Q., Yang Y., Chen F., Ge W., Wu H., Liu Z. (2018). XIST Derepression in Active X Chromosome Hinders Pig Somatic Cell Nuclear Transfer. Stem Cell Rep..

[16] Lehnertz B., Ueda Y., Derijck A.A., Braunschweig U., Perez-Burgos L., Kubicek S., Chen T., Li E., Jenuwein T., Peters A.H. (2003). Suv39h-mediated histone H3 lysine 9 methylation directs DNA methylation to major satellite repeats at pericentric heterochromatin. Curr. Biol. CB.

[17] Vire E., Brenner C., Deplus R., Blanchon L., Fraga M., Didelot C., Morey L., Van Eynde A., Bernard D., Vanderwinden J.M. (2006). The Polycomb group protein EZH2 directly controls DNA methylation. Nature.

[18] Hauser D., Weber H.P., Sigg H.P. (1970). Isolation and configuration of Chaetocin. Helv. Chim. Acta.

[19] Sekita S., Yoshihira K., Natori S., Udagawa S., Muroi T., Sugiyama Y., Kurata H., Umeda M. (1981). Mycotoxin production by Chaetomium spp. and related fungi. Can. J. Microbiol..

[20] Greiner D., Bonaldi T., Eskeland R., Roemer E., Imhof A. (2005). Identification of a specific inhibitor of the histone methyltransferase SU(VAR)3-9. Nat. Chem. Biol..

[21] Liu X., Guo S., Liu X., Su L. (2015). Chaetocin induces endoplasmic reticulum stress response and leads to death receptor 5-dependent apoptosis in human non-small cell lung cancer cells. Apoptosis Int. J. Program. Cell Death.

[22] Lai Y.S., Chen J.Y., Tsai H.J., Chen T.Y., Hung W.C. (2015). The SUV39H1 inhibitor chaetocin induces differentiation and shows synergistic cytotoxicity with other epigenetic drugs in acute myeloid leukemia cells. Blood Cancer J..

[23] Chaib H., Nebbioso A., Prebet T., Castellano R., Garbit S., Restouin A., Vey N., Altucci L., Collette Y. (2012). Anti-leukemia activity of chaetocin via death receptor-dependent apoptosis and dual modulation of the histone methyl-transferase SUV39H1. Leukemia.

[24] Isham C.R., Tibodeau J.D., Jin W., Xu R., Timm M.M., Bible K.C. (2007). Chaetocin: A promising new antimyeloma agent with in vitro and in vivo activity mediated via imposition of oxidative stress. Blood.

[25] Lee Y.M., Lim J.H., Yoon H., Chun Y.S., Park J.W. (2011). Antihepatoma activity of chaetocin due to deregulated splicing of hypoxia-inducible factor 1alpha pre-mRNA in mice and in vitro. Hepatology.

[26] Giraldo A.M., Ball S., Bondioli K.R. (2012). Production of transgenic and knockout pigs by somatic cell nuclear transfer. Methods Mol. Biol..

[27] Watanabe M., Kurome M., Matsunari H., Nakano K., Umeyema K., Shiota A., Nakauchi H., Nagashima H. (2012). The creation of transgenic pigs expressing human proteins using BAC-derived, full-length genes and intracytoplasmic sperm injection-mediated gene transfer. Transgenic Res..

[28] Dean W., Santos F., Stojkovic M., Zakhartchenko V., Walter J., Wolf E., Reik W. (2001). Conservation of methylation reprogramming in mammalian development: Aberrant reprogramming in cloned embryos. Proc. Natl. Acad. Sci. USA.

[29] Zheng H., Huang B., Zhang B., Xiang Y., Du Z., Xu Q., Li Y., Wang Q., Ma J., Peng X. (2016). Resetting Epigenetic Memory by Reprogramming of Histone Modifications in Mammals. Mol. Cell.

[30] Allis C.D., Jenuwein T. (2016). The molecular hallmarks of epigenetic control. Nat. Rev. Genet..

[31] Shi L., Wu J. (2009). Epigenetic regulation in mammalian preimplantation embryo development. Reprod. Biol. Endocrinol. RB&E.

[32] Zhang Y.M., Gao E.E., Wang Q.Q., Tian H., Hou J. (2018). Effects of histone methyltransferase inhibitor chaetocin on histone H3K9 methylation of cultured ovine somatic cells and development of preimplantation cloned embryos. Reprod. Toxicol..

[33] Liu X., Wang Y., Gao Y., Su J., Zhang J., Xing X., Zhou C., Yao K., An Q., Zhang Y. (2018). H3K9 demethylase KDM4E is an epigenetic regulator for bovine embryonic development and a defective factor for nuclear reprogramming. Development.

[34] Weng X.G., Cai M.M., Zhang Y.T., Liu Y., Liu C., Liu Z.H. (2019). Improvement in the in vitro development of cloned pig embryos after kdm4a overexpression and an H3K9me3 methyltransferase inhibitor treatment. Theriogenology.

[35] Jeong Y.I., Park C.H., Kim H.S., Jeong Y.W., Lee J.Y., Park S.W., Lee S.Y., Hyun S.H., Kim Y.W., Shin T. (2013). Effects of Trichostatin A on In vitro Development of Porcine Embryos Derived from Somatic Cell Nuclear Transfer. Asian-Australas. J. Anim. Sci..

[36] Cordova A., King W.A., Mastromonaco G.F. (2017). Choosing a culture medium for SCNT and iSCNT reconstructed embryos: From domestic to wildlife species. J. Anim. Sci. Technol..

[37] Huang J., Zhang H., Yao J., Qin G., Wang F., Wang X., Luo A., Zheng Q., Cao C., Zhao J. (2016). BIX-01294 increases pig cloning efficiency by improving epigenetic reprogramming of somatic cell nuclei. Reproduction.

[38] Zhai Y., Zhang Z., Yu H., Su L., Yao G., Ma X., Li Q., An X., Zhang S., Li Z. (2018). Dynamic Methylation Changes of DNA and H3K4 by RG108 Improve Epigenetic Reprogramming of Somatic Cell Nuclear Transfer Embryos in Pigs. Cell. Physiol. Biochem..

[39] Zhang Z., Zhai Y., Ma X., Zhang S., An X., Yu H., Li Z. (2018). Down-Regulation of H3K4me3 by MM-102 Facilitates Epigenetic Reprogramming of Porcine Somatic Cell Nuclear Transfer Embryos. Cell. Physiol. Biochem..

[40] Zhao C., Shi J., Zhou R., He X., Yang H., Wu Z. (2018). DZNep and UNC0642 enhance in vitro developmental competence of cloned pig embryos. Reproduction.

[41] Erhardt S., Su I.H., Schneider R., Barton S., Bannister A.J., Perez-Burgos L., Jenuwein T., Kouzarides T., Tarakhovsky A., Surani M.A. (2003). Consequences of the depletion of zygotic and embryonic enhancer of zeste 2 during preimplantation mouse development. Development.

[42] Chen P., Yao J.F., Huang R.F., Zheng F.F., Jiang X.H., Chen X., Chen J., Li M., Huang H.F., Jiang Y.P. (2015). Effect of BIX-01294 on H3K9me2 levels and the imprinted gene Snrpn in mouse embryonic fibroblast cells. Biosci. Rep..

[43] Salimi M., Shirazi A., Norouzian M., Mehrazar M.M., Naderi M.M., Shokrgozar M.A., Omrani M., Hashemi S.M. (2020). Histone Modifications of H3K4me3, H3K9me3 and Lineage Gene Expressions in Chimeric Mouse Embryo. Cell J..

[44] Iwasa E., Hamashima Y., Fujishiro S., Higuchi E., Ito A., Yoshida M., Sodeoka M. (2010). Total synthesis of (+)-chaetocin and its analogues: Their histone methyltransferase G9a inhibitory activity. J. Am. Chem. Soc..

[45] Tran H.T., Kim H.N., Lee I.K., Nguyen-Pham T.N., Ahn J.S., Kim Y.K., Lee J.J., Park K.S., Kook H., Kim H.J. (2013). Improved therapeutic effect against leukemia by a combination of the histone methyltransferase inhibitor chaetocin and the histone deacetylase inhibitor trichostatin A. J. Korean Med. Sci..

[46] Chiba T., Saito T., Yuki K., Zen Y., Koide S., Kanogawa N., Motoyama T., Ogasawara S., Suzuki E., Ooka Y. (2015). Histone lysine methyltransferase SUV39H1 is a potent target for epigenetic therapy of hepatocellular carcinoma. Int. J. Cancer.

[47] Sulewska A., Niklinska W., Kozlowski M., Minarowski L., Naumnik W., Niklinski J., Dabrowska K., Chyczewski L. (2007). DNA methylation in states of cell physiology and pathology. Folia Histochemica et Cytobiologica.

[48] Ge Y.Z., Pu M.T., Gowher H., Wu H.P., Ding J.P., Jeltsch A., Xu G.L. (2004). Chromatin targeting of de novo DNA methyltransferases by the PWWP domain. J. Biol. Chem..

[49] Tahiliani M., Koh K.P., Shen Y., Pastor W.A., Bandukwala H., Brudno Y., Agarwal S., Iyer L.M., Liu D.R., Aravind L. (2009). Conversion of 5-methylcytosine to 5-hydroxymethylcytosine in mammalian DNA by MLL partner TET1. Science.

[50] Piccolo F.M., Fisher A.G. (2014). Getting rid of DNA methylation. Trends Cell Biol..

[51] Enright B.P., Kubota C., Yang X., Tian X.C. (2003). Epigenetic characteristics and development of embryos cloned from donor cells treated by trichostatin A or 5-aza-2′-deoxycytidine. Biol. Reprod..

[52] Mizushima N., Komatsu M. (2011). Autophagy: Renovation of cells and tissues. Cell.

[53] Song B.S., Yoon S.B., Kim J.S., Sim B.W., Kim Y.H., Cha J.J., Choi S.A., Min H.K., Lee Y., Huh J.W. (2012). Induction of autophagy promotes preattachment development of bovine embryos by reducing endoplasmic reticulum stress. Biol. Reprod..

[54] Chi D., Zeng Y., Xu M., Si L., Qu X., Liu H., Li J. (2017). LC3-Dependent Autophagy in Pig 2-Cell Cloned Embryos Could Influence the Degradation of Maternal mRNA and the Regulation of Epigenetic Modification. Cell. Reprogramming.

[55] Cho Y.H., Han K.M., Kim D., Lee J., Lee S.H., Choi K.W., Kim J., Han Y.M. (2014). Autophagy regulates homeostasis of pluripotency-associated proteins in hESCs. Stem Cells.

[56] Chen T., Shen L., Yu J., Wan H., Guo A., Chen J., Long Y., Zhao J., Pei G. (2011). Rapamycin and other longevity-promoting compounds enhance the generation of mouse induced pluripotent stem cells. Aging Cell.

[57] Shen X., Zhang N., Wang Z., Bai G., Zheng Z., Gu Y., Wu Y., Liu H., Zhou D., Lei L. (2015). Induction of autophagy improves embryo viability in cloned mouse embryos. Sci. Rep..

[58] Jung H.J., Seo I., Casciello F., Jacquelin S., Lane S.W., Suh S.I., Suh M.H., Lee J.S., Baek W.K. (2016). The anticancer effect of chaetocin is enhanced by inhibition of autophagy. Cell Death Dis..

[59] Liao X., Fan Y., Hou J., Chen X., Xu X., Yang Y., Shen J., Mi P., Huang X., Zhang W. (2019). Identification of Chaetocin as a Potent non-ROS-mediated Anticancer Drug Candidate for Gastric Cancer. J. Cancer.

[60] Jeong P.S., Yoon S.B., Choi S.A., Song B.S., Kim J.S., Sim B.W., Park Y.H., Yang H.J., Mun S.E., Kim Y.H. (2017). Iloprost supports early development of in vitro-produced porcine embryos through activation of the phosphatidylinositol 3-kinase/AKT signalling pathway. Reprod. Fertil. Dev..

[61] Richter A., Kurome M., Kessler B., Zakhartchenko V., Klymiuk N., Nagashima H., Wolf E., Wuensch A. (2012). Potential of primary kidney cells for somatic cell nuclear transfer mediated transgenesis in pig. BMC Biotechnol..

