# Supplementary material for: Chaetocin Improves Pig Cloning Efficiency by Enhancing Epigenetic Reprogramming and Autophagic Activity
Source: Int J Mol Sci. 2020 Jul 8;21(14):4836. doi: 10.3390/ijms21144836 (PMC7402317; doi:10.3390/ijms21144836)
Supplement: Supplementary file 1 [file ijms-21-04836-s001.pdf]

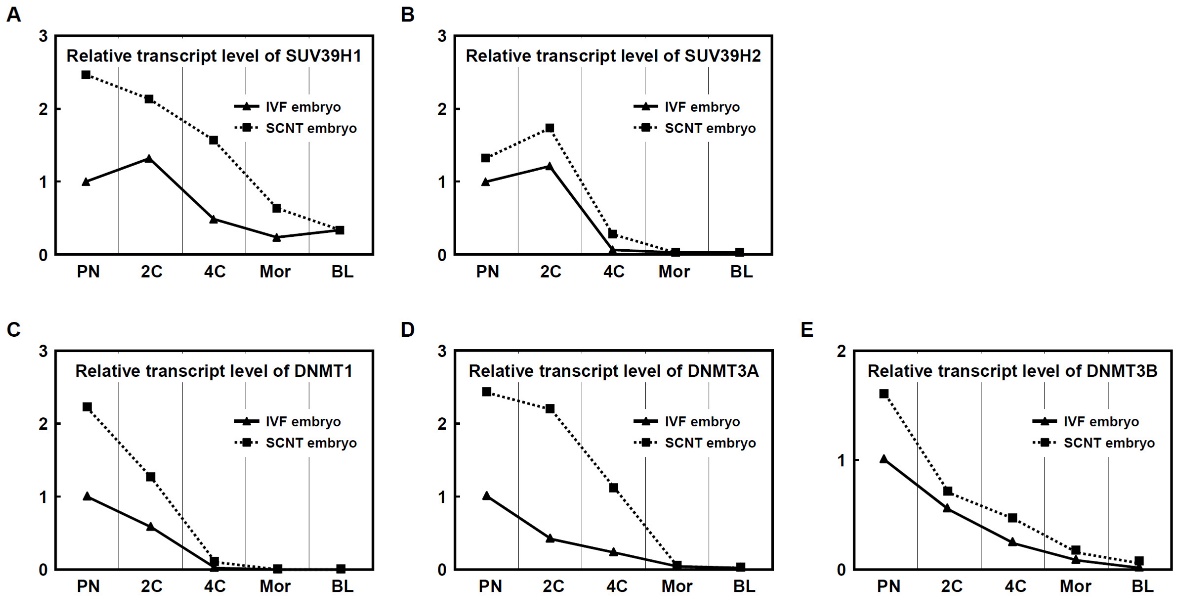

Supplementary Figure 1

Figure S1. mRNA levels of the H3K9me3-specific methyltransferases (A) *suv39h1* and (B) *suv39h2* and the DNMTs (C) *DNMT1*, (D) *DNMT3a*, and (E) *DNMT3b* during preimplantation development of porcine IVF and SCNT embryos. The data are from three independent experiments and are means ± SEM (**P* < 0.05).

Supplementary table S1 Primer sequences for qRT-PCR

Gene	Primer sequences	GenBank accession no.	Product size (bp)
Suv39h1	F: 5'- GGG CTT TGT CAA CCA CAG TT -3'<br>R: 5'- TCA GTC CCA CAC TTG CAC TC -3'	XM_013992210.1	165
Suv39h2	F: 5'- CCGGGAATCAGCTTAGTCAA -3'<br>R: 5'- GGGGTACCTGGTGGAATTTT -3'	NM_001039747.1	140
DNMT1	F: 5'- AGG ACC GGA TCT CTT GGA TT -3'<br>R: 5'- AGA GCT GTG ACC CTG GCT AA -3'	NM_001032355.1	163
DNMT3a	F: 5'- CCT GGA ACA CCC TCT CTT CA -3'<br>R: 5'- CAG CAG ATG GTG CAG TAG GA -3'	NM_001097437.1	114
DNMT3b	F: 5'- GAC CAG TCT TCC GAC TCC AA -3'<br>R: 5'- CTC CCT CTT GGA CAG TCG AG -3'	NM_001348900.1	108
Oct4	F: 5'- AGT GAG AGG CAA CCT GGA GA -3'<br>R: 5'- ACT GCT TGA TCG TTT GCC CT -3'	NM_001113060.1	151
Nanog	F: 5'- GGT TCC AGA ACC AGC GAA TGA -3'<br>R: 5'- CTG TAC TGG CTG AGC CCT GA -3'	XM_021092390.1	93
Sox2	F: 5'- AGC CCA GAC CGA GTT AAG CG -3'<br>R: 5'- TGG GGT TCT CTT GGG CCA TC -3'	NM_001123197.1	85
Cdx2	F: 5'- GGC AGC CAA GTG AAA ACC AG -3'<br>R: 5'- GCC TTT CTC CGA ATG GTG AT -3'	NM_001278769.1	119
Bax	F: 5'- CGA TCT CGA AGG AAG TCC AG -3'<br>R: 5'- AAG CGC ATT GGA GAT GAA CT -3'	XM_003127290.5	251
Bak	F: 5'- CTA GAA CCT AGC AGC ACC AT -3'<br>R: 5'- CGA TCT TGG TGA AGT ACT C -3'	XM_001928147.3	151
Bcl-xl	F: 5'- AGG GCA TTC AGT GAC CTG AC -3'<br>R: 5'- TGG ATC CAA GGC TCT AGG TG -3'	NM_214285.1	242
Bcl2	F: 5'- GGA GGG GAC ACT CTT CTT CC -3'<br>R: 5'- CTG GGC ACA ATT GGT AGC TT -3'	XM_021099593.1	189
Beclin1	F: 5'- AGG AGC TGC CGT TGT ACT GT -3'<br>R: 5'- TGT CTC GCC TTT CTC AAC CT -3'	NM_001044530.1	125
ATG7	F: 5'- CTG TGA CTG TGT CGG AGG AC -3'<br>R: 5'- CCC ATA GCT GCT GCC ATT AT -3'	NM_001190285.1	116
LC3	F: 5'- CCG AAC CTT CGA ACA GAG AG -3'<br>R: 5'- AGG CTT GGT TAG CAT TGA GC -3'	NM_001190290.1	206

BMP15	F: 5'- TGG TGA GGC CAT TGG TTA AT -3'<br>R: 5'- AGA GGT GGA AGG GAG CTA GG -3'	NM_001005155.2	156
GDF9	F: 5'- AAC ACT GTC CGG CTC TTC AC -3'<br>F: 5'- CCA GGC TGC ACT CAC ATT TA -3'	NM_001001909.1	202
DPPA3	F: 5'- CCG GAC TCA GGA TTC TCA AA -3'<br>F: 5'- CGG TTG AGG TCG ATT TTC TG -3'	XM_021093127.1	160
C-mos	F: 5'- GGG AGC AAC TGA ACT TGG AG -3'<br>R: 5'- AGA ATG TTC GCT GGC TTC AG -3'	NM_001113219.1	115
H100	F: 5'- GAA GGC AAA GGT CAA AGC AG -3'<br>R: 5'- AGG GAT CTT GTT CCC CAT CT -3'	NM_001205063.1	127
ZAR-1	F: 5'- CCT GCG CTT CCA GTT CTT AG -3'<br>R: 5'- TGT TAG TGC CCT GGA CAC AC -3'	NM_001129956.1	104
GAPDH	F: 5'- CCC TGA GAC ACG ATG GTG AA -3'<br>R: 5'- GGA GGT CAA TGA AGG GGT CA -3'	NM_001206359.1	147

Supplementary Table 2. Effect of chaetocin concentration on the early development of porcine SCNT embryos

Chaetocin (nM)	No. of embryos examined	Cleavage (%)	Blastocyst (%)	Hatching rate (%)	Total cell number
0	148	122 (81.8±0.4)[a]	36 (24.3±1.1)[a]	8 (5.4±1.0)[a]	31.4±1.7[a]
0.1	149	123 (82.5±1.8)[a,b]	41 (27.5±0.9)[a]	18 (12.1±1.8)[a,b]	37.5±2.1[a,b]
0.5	148	128 (87.1±0.8)[b]	52 (35.1±1.5)[b]	26 (17.6±1.7)[b]	40.5±3.0[b]
1	149	119 (79.8±1.2)[a]	36 (24.1±2.7)[a]	12 (8.0±1.8)[a]	35.7±1.9[a,b]

Data are the mean ± SEM, and values with different superscript letter within a column differ significantly ($p < 0.05$).

Supplementary Table 3. Effect of duration of treatment with 0.5 nM chaetocin on the early development of porcine SCNT embryos

Chaetocin (h)	No. of embryos examined	Cleavage (%)	Blastocyst (%)	Hatching rate (%)	Total cell number
0	132	100 (75.2±1.4)[a]	29 (22.2±1.4)[a]	7 (5.2±1.1)[a,c]	30.4±1.4[a]
24	132	109 (84.9±1.4)[b]	44 (33.6±1.2)[b]	22 (16.7±1.3)[b]	40.8±1.4[b]
48	132	99 (74.5±2.6)[a]	28 (21.2±1.9)[a]	13 (9.6±1.7)[c]	34.8±1.9[a]
72	132	95 (71.5±3.0)[a]	17 (12.9±1.6)[c]	3 (2.2±0.7)[a]	30.5±1.5[a]

Data are the mean ± SEM, and values with different superscript letter within a column differ significantly ($p < 0.05$).

Supplementary Table 4. Effect of chaetocin on the ICM/TE ratio in porcine SCNT blastocysts

Groups	No. of blastocysts examined	No. of nuclei			ICM/TE (%)
		ICM	TE	Total	
Con	20	6.1±0.4[a]	26.5±1.2[a]	32.6±1.4[a]	23.5±1.7
Chaetocin	20	8.7±0.4[b]	33.6±1.1[b]	42.3±1.1[b]	26.5±1.5

Data are the mean ± SEM, and values with different superscript letter within a column differ significantly ($p < 0.05$).

Supplementary Table 5. Effect of chaetocin on cell survival in porcine SCNT blastocysts

Groups	No. of blastocysts examined	No. of TUNEL-positive cells	Apoptosis (%)
Con	21	2.9±0.4[a]	8.6±1.2[a]
Chaetocin	21	1.6±0.3[b]	3.7±0.7[b]

Data are the mean ± SEM, and values with different superscript letter within a column differ significantly (p < 0.05).
